# A Hybrid Hand-Crafted and Deep Neural Spatio-Temporal EEG Features Clustering Framework for Precise Emotional Status Recognition

**DOI:** 10.3390/s22145158

**Published:** 2022-07-09

**Authors:** Qazi Mazhar ul Haq, Leehter Yao, Wahyu Rahmaniar, Fawad, Faizul Islam

**Affiliations:** 1 Department of Electrical Engineering, National Taipei University of Technology, Taipei 106, Taiwan; qazi@ntut.edu.tw (Q.M.u.H.); wahyu@ntut.edu.tw (W.R.); 2 Department of Telecommunication Engineering, University of Engineering and Technology Taxila, Rawalpindi 47050, Punjab, Pakistan; engr.fawad@students.uettaxila.edu.pk; 3 School of Automation, Nanjing University of Science and Technology, Nanjing 210094, China; faiz1991@njust.edu.cn

**Keywords:** differential entropy, emotion status recognition, spatio-temporal features, hybrid model

## Abstract

Human emotions are variant with time, non-stationary, complex in nature, and are invoked as a result of human reactions during our daily lives. Continuously detecting human emotions from one-dimensional EEG signals is an arduous task. This paper proposes an advanced signal processing mechanism for emotion detection from EEG signals using continuous wavelet transform. The space and time components of the raw EEG signals are converted into 2D spectrograms followed by feature extraction. A hybrid spatio-temporal deep neural network is implemented to extract rich features. A differential-based entropy feature selection technique adaptively differentiates features based on entropy, based on low and high information regions. Bag of Deep Features (BoDF) is applied to create clusters of similar features and computes the features vocabularies for reduction of feature dimensionality. Extensive experiments are performed on the SEED dataset, which shows the significance of the proposed method compared to state-of-the-art methods. Specifically, the proposed model achieved 96.7%, 96.2%, 95.8%, and 95.3% accuracy with the SJTU SEED dataset, for SVM, ensemble, tree, and KNN classifiers, respectively.

## 1. Introduction

Films are a form of visual art that can easily evoke human emotions. Depending on the human being’s conditions and circumstances, they feel different emotions, for example, human beings in a positive mood are more likely to feel happy emotions [1]. Effective indexing in multimedia can enhance multimedia restoration, and make recommendations for evoking multiple emotions [2]. It is a convenient way to describe the traces and feelings of human emotions without disturbing other human beings. These traces are beneficial, since there is no need for self-reporting affected by different social and personality factors. The emotional behavior of every human is recorded through electroencephalogram signals (EEG). The instant these emotional profiles or traces are felt, a signal is shown on the EEG, and this has applications in video indexing and summarization [3]. There are different types of emotions, which are categorized into three categories. One is expressed emotions that are real human emotion, another is felt emotions, and last one is the expected emotions. Expressed emotions are those that artists or creators try to present, irrespective of whether they are actually feeling those emotions. Expected emotions are those felt by the audience when watching the video content. Felt emotions are the personal feelings that are felt by the audience members individually. This paper aims to identify felt emotions using EEG signals extracted while watching multiple emotion-evoking movies.

**Figure 1:**
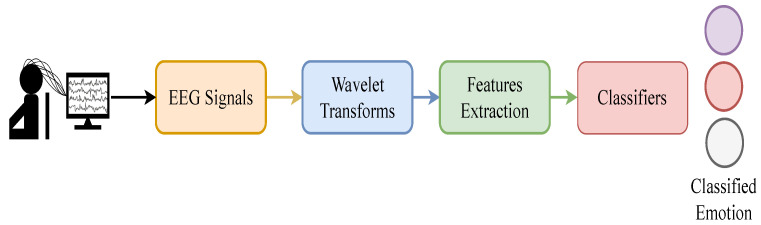
Overview of the proposed framework.

The feelings of human beings can be changed according to the responses the person has to external and internal means that effect and alter human emotions. Most of the existing research detected human emotions using non-physiological signals, such as speech, body movement, facial expressions, along with physiological signals, such as functional magnetic resonance imaging (fMRI), skin resistance, electrocardiogram, and EEG [4,5,6,7,8]. Physiological signals, in comparison to non-physiological signals, are not influenced by the environment, especially EEG signals, due to their repeatability and reproducibility. Various methods were implemented in the literature to evaluate EEG signals for emotion detection. Traditionally, researchers performed an evaluation on four different models for emotion detection, such as discrete, dimension, processing, and temporal models [9]. The discrete emotion model considers multiple emotions and measures them on a scale based on the differential measurement of emotion [10]. The dimension model is based on the consciousness of the human being and considers valence, arousal, and dominance [11]. Temporal models measure emotions based on duration, such as short term, medium, and long term emotions [12]. In addition, the processing level models measure emotions at different levels, such as low-level, which invokes effective signal and motor actions, mid level and high level emotions [13]. Time-frequency analysis is performed to obtain various characteristics because it provides salient information about the emotional states. A differential entropy is measured for various frequency bands associated with these EEG signals for emotion assessments through beta and gamma rhythms. Using spatial-temporal recurrent neural networks (STRNN), the time-frequency domain features of EEG signals were categorized into 18 multi-linear features information by a spatial and temporal dependent model [14]. A collection of multiple emotional statuses from EEG signal analysis through different channels is used for cross domain assessment of emotion [15]. A recursive emotion feature elimination (RFE) method is used to reduce the repetitive features dimension. However, the problem of emotion assessment in these methods is approached in two ways. Either the researchers used the channels that provided high accuracy but required high computations or reduced the channels, which increased the computational speed but compromised the accuracy results. In this article, an EEG-based emotion assessment model is proposed, which uses differential entropy-based channel selection (DECS) for selecting channels that provide high-quality features and bag of deep features (BoDF), which further reduces the feature dimension. A 2-D spectrogram is obtained by taking the CWT of the pre-processed EEG emotion dataset. The GoogleNet model, which takes the 2-D spectrogram as input, provides us with the feature vector of all the subjects. The DECS algorithm allows us to select the channels which provide high entropy features. The dimensions of the high-quality features are reduced using BoDF by k-means clustering, and the visual vocabulary is generated. The visual vocabulary is then fed to the multi-class classifiers, which assesses the emotions using all kernels to classify three states of the SEED dataset: neutral, positive, and negative emotions. The overflow of the proposed emotion detection scheme is described in Figure 1.

The reminder of the paper is organized as follows: Section 2 presents previous work performed on EEG signals for emotion detection. Section 3 presents the formulation of the proposed emotion detection framework. Section 4 illustrates experimental results and an evaluation, and makes a comparison with previous methods. Section 5 concludes the paper and provides further research directions for emotion detection.

## 2. Related ork

Emotion detection gained a lot of attention in the last few years due to its direct links with multiple fields such as psychology, cognitive sciences, computer science, life sciences, physiology, and marketing [16,17]. There are a lot of techniques proposed to extract EEG signals from the brain and process them through computer-aided design to detect human emotions. For the last few decades, researchers have been trying to develop different methods for the human being’s brain–computer interface to collect emotional data. The brain–computer interface is used to control various devices and actions by brain signals [18]. Multiple human emotions are felt while watching movies, and the corresponding brain signals are recorded for further investigation. These brain signals are categorized and recognized through EEG signals. The EEG signals are retrieved from the brain with an electrode placed on the skull [19]. Although numerous researchers have attempted emotion detection through EEG signals, the detection process still requires much improvement and better measurement. A normalization of common spatial patterns (CSP) [20] is helpful in the reduction of noise and artifacts. An attempt at real-time emotion detection is achieved through virtual reality and the Internet of Things, which are used to provide users with a virtual and physical environment [21]. A classification framework is used that applies optimal and learning filter ratio criterion for emotion detection [22]. The optimal filters are used that helps in the automatic understanding of data. Most of the previous methods use time-frequency analysis and sparse distribution, namely discrete wavelet transform [23] and Fourier transform [19]. However, the emotional states are subjective and complex in nature.

Recent studies used machine learning tools due to the development and advancement in machine learning techniques for classification and detection tasks [24]. Various machine learning techniques were proposed to analyze and detect human emotion from EEG signals. The selection process of channels and features is critical for accurate detection. The computational cost tends to be high if there is a large dataset and vast training data. Different techniques were proposed for object detection and segmentation through deep learning classifiers [25,26]. The decomposition is performed with multiple traditional time-frequency decomposition methods, such as empirical mode decomposition (EMD) and discrete wavelet transform (DWT) to fragment the signals. Similar techniques, such as naive bayes, k-nearest neighbors, and multi-layers perceptron are applied to extract 13 different facial expressions [27]. Various feature extraction methods were used in the literature to improve the performance on emotion detection [28,29,30]. These feature extraction methods are based on extracting and measuring the dimension of facial landmarks. They use multi-model learning of Electromyography (EMG) and EEGs to conceive and extract information from the brain, and other sources of extracting information are texts, images, and videos. However, it is complicated to decipher the information retrieved from these images, texts, and videos. A differential entropy is measured from the respective EEG pulses where gamma and beta are applied as informative pulses to improve the learning of EEG signals for emotion detection [14]. In total, 18 different linear and non-linear vibrations through spatio-temporal neural networks were collected. Additionally, a database of EEG signals and emotion analysis was proposed for cross-target facial emotion assessment [15]. Motor imagery classification (MIC) used Hilbert transform and wavelet decomposition in combination with CapsNets backbone to achieve a better understanding of the EEG signals [25]. Cross-subject generalizability was accomplished through flexible analytic wavelet transform with machine learning classifiers [31]. A novel natural language processing was implemented for emotion detection through sentiment analysis [32]. A survey was performed on using sentiment analysis on social media data and presented a new direction in predicting human behavior [33]. Similarly sentiment analysis was applied to obtain human experiences such as finding the obesity rate by observing twitter data [34], investigation of tourist attractions [35], twitter sentiment and expressions [36], and overall happiness and patterns of life [37]. Multi-model learning (MM) with a bag of deep features is applied with machine learning classifiers using all the data collection channels for emotion detection [38]. A comparison of these methods and a corresponding number of channels used and backbone network is presented in Table 1. However, detecting emotions through EEG signals is quite challenging due to its undefined margins and boundaries. Traditionally, the assessment of EEG signals in these methods is approached in two ways; either using a high frequency that increases computational complexity or reduced channels that drop the accuracy. Furthermore, these methods are expensive in terms of cost and installation, making them unstable and infeasible in real-world situations. The difficult task is to design a novel method for detecting multiple emotions. The frequency components tend to change with respect to time, so the information is inadequate for detecting human emotions. Therefore, this paper uses a continuous wavelet transform (CWT) to study non-stationary EEG signals.

## 3. Proposed Methodology

**Figure 2:**
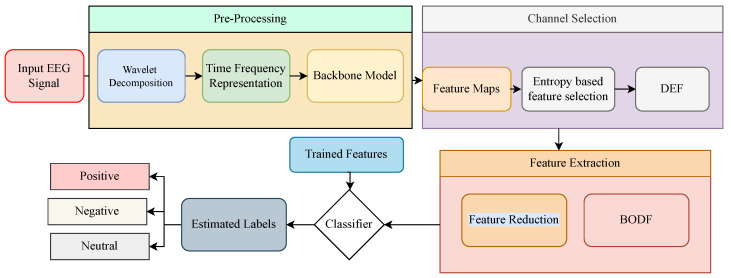
The proposed framework for classification of Emotions form the EEG signals of the SEED Dataset.

This section presents the proposed architecture for emotion detection through EEG signals. The block diagram of the proposed method is illustrated in Figure 2. For sparse representation, the EEG signals are passed by continuous wavelet transform. GoogleNet further processes the sparsed signals for feature extraction. These features are divided based on the entropy of every feature through differential entropy-based feature selection. The redundant features are reduced by the BoDF and further classified by SVM, KNN, decision trees, and ensemble networks classifiers, resulting in a classified emotion. Each section of the proposed method is explained below.

### 3.1. Decomposition into Time-Frequency Spectrum

In real-life situations, the behavior of emotional states are subjective, complex, and non-stationary. To localize the subject of interest, time-frequency analysis is implemented to present sparse representation. The traditional way to transform the EEG signals x(t) to spectrograms is through short-time Fourier transform (STFT) $X_{stft}\left(\tau ,\omega \right)$, where τ, ωϵR, τ≠0, and R is the set of real number. The $X_{stft}\left(\tau ,\omega \right)$ transforms x(t) through window function w(t), such as

(1) $$X_{stft}\left(\tau ,\omega \right)=\int_{-\infty }^{\infty }x\left(t\right)\left(w\left(\tau -t\right)\right)e^{-j\omega t}dt.$$

As the short window size of STFT results in more concise and provide sufficient understanding for time but ultimately degrading frequency information. However, a bigger window size has the opposite effect to a shorter window. To achieve a better analysis for both time and frequency domain, we applied continues wavelet analysis to extract necessary features and develop an image of the one-dimensional (1D) EEG signals. The continues wavelet analysis provides a better relationship between the decomposed version and the parent wavelet.

The emotional states are continuous and the frequency components ω tends to change with respect to time *t*. To present a sparser representation, CWT $W_{x}\left(m,n\right)$ is used to provide further segments of the $X_{stft}\left(\tau ,\omega \right)$, where *m* and *n* are the translating and scaling parameter of $\psi \left(t\right)$, respectively. If u(t) is the EEG signal in *t*, CWT transformation can be defined as

(2) $$W_{x}\left(m,n\right)=\frac{1}{\sqrt{\left|m\right|}}\int_{-\infty }^{\infty }u\left(t\right)\psi \left(\frac{t-n}{m}\right)dt.$$

where $\psi \left(t\right)$ is the wavelet function called “mother wavelet” depending on scale value m>0, *a*ϵR and translation value n>0, *n*ϵR. *m* and *n* are normalized such that they stay at the same level for different values with the function of $\frac{1}{\sqrt{\left|m\right|}}$. The scaling factor, *a*, controls the compression and expansion of the time but have reciprocal effect on frequency. This benefits in recognizing the periodical trends in input signal. Whereas the shifting factor, *b*, translates multidimensional decomposed components of the original signal u(t). Continues wavelet analysis has the ability to recognize the abrupt changes in the frequency for multi-scales of the EEG signal. The decomposed wavelet at the desired time can obtained through applying the translated versions $\psi \left(t\right)$ of wavelets to the original mother wavelet.

CWT provides an excellent opportunity to extract and investigate complicated spectral features of a signal. Function $\psi \left(t\right)$ is a continuous in time and frequency function which is used to provide “daughter wavelets” for each possible time-translation and time-scaling as follows,

(3) $$\psi_{m,n}\left(t\right)=\frac{1}{\sqrt{\left|m\right|}}\psi \left(\frac{t-n}{m}\right).$$

To reconstruct the original signal, inverse continues wavelet transform (ICWT) can represented as double-integral form, which used Morse wavelet and L1 normalization as follows,

(4) $$u\left(t\right)=\frac{1}{C_{\Psi }}\int_{-\infty }^{\infty }\int_{-\infty }^{\infty }\frac{1}{\sqrt{\left|m\right|}}W_{x}\left(m,n\right)\hat{\psi }\left(\frac{t-n}{m}\right)\frac{dbdm}{m^{2}},$$

where $\hat{\psi }\left(t\right)$ is the dual function of $\psi \left(t\right)$, $\hat{\psi }$ϵ$L^{2}R$, $C_{\Psi }$ is admissible wavelet with value in range of [0,∞], can be defined as,

(5) $$C_{\psi }=\int_{-\infty }^{\infty }\frac{\tilde{\hat{\psi }}\left(\omega \right)}{\left|\omega \right|}d\omega .$$

where $\psi \left(\omega \right)$ is the Fourier transform of $\psi \left(t\right)$ and $\tilde{\hat{\psi }}=\hat{\psi }\left(\omega \right)\psi \left(\omega \right)$. The function $C_{\psi }$ has to be integrate to zero and oscillatory. This admissibility condition is used to analyze and reconstruct a signal without loss of information. Then, the mother wavelet, $\psi \left(t\right)$ is reconstructed through second inverse wavelet, such as

(6) $$u\left(t\right)=\frac{1}{2\pi \tilde{\hat{\psi }}}\int_{-\infty }^{\infty }\int_{-\infty }^{\infty }\frac{1}{m^{2}}W_{u}\left(m,n\right)e^{\left(\frac{i\left(t-n\right)}{m}\right)}dbdm.$$

The wavelets in time domain can be obtained as,

(7) $$\psi \left(t\right)=\omega \left(t\right)e^{it},$$

where $\omega \left(t\right)$ represents a window with a finite number of points. The low and high pass filter banks in continuous wavelet transform represent the low and high-frequency distribution in decomposition and reconstruction stages according to time. The translated and scaled decomposed versions of wavelets are moved in time across the original signal that results in estimating coefficients for multiple scales. The transformation of each recording of EEG signal of siz 1×i are transformed to a two-dimensional matrix of size i×j, where *j* is number of multiple scales and *i* is whole length of the signal.

**Figure 3:**
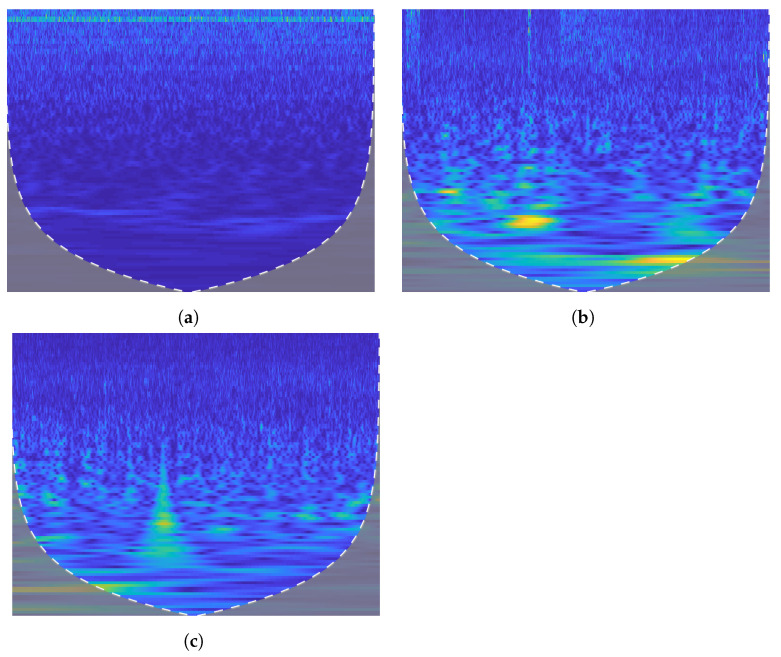
Time frequency distribution for three classes of SEED dataset. (**a**) Neutral. (**b**) Positive. (**c**) Negative.

Furthermore, the feature vectors are extracted from the two-dimensional matrix. These feature vectors are combined to obtain the final feature matrix of size p×q where *p* represnts the total number of recordings and *q* represents total number of features. In the feature matrix p×q, the recordings are divided into three target emotions for each EEG data: neutral, positive, and negative, as shown in Figure 3. The effect of wavelet transform is visualized in Figure 3 that represents the time versus frequency distribution of different classes in the SEED dataset.

### 3.2. Feature Extraction

To provide enough features for learning the whole structure of spectrograms, GoogleNet inception model [40] is proposed as our backbone model for feature extraction. The obtained spectrum images from continuous wavelet transform is trained by a pre-trained GoogleNet model. This model is originally implemented on the ImageNet dataset, consisting of 1000 classes. The linear output layers consist of 1000 hidden nodes. The architecture consists of 22 deep layers and nine inception modules, while two max-pooling layers are used between every inception layer. The core idea of the inception module is to find the optimal sparse structure from provided coarse components. The final softmax layer is removed, and multiple traditional classifiers are applied to perform classification.

**Figure 4:**
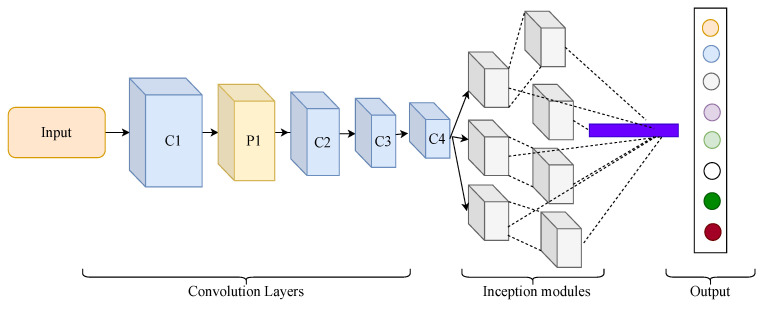
The overview of backbone GoogleNet model.

The whole architecture of the GoogleNet model is illustrated in Figure 4. The basic unit of GoogleNet achieves better accuracy and can parameters can be easily changed or modified in the model. We modified the basic architecture for SEED dataset. The input image is resized to 224 × 224 RGB color space with zero mean value. Each convolution layer is comprised of filters that are convolved with the input data to extract features. During the first layer, input image of size 224 × 224 is reduced to a dimension of 112 × 112 by a convolution filter of 34 × 34 and stride of 2. The formula for first layer operation is as follows,

(8) $$Y_{out}=\frac{Y_{in}+2p-k}{s}+1,$$

where $Y_{in}$ is input size, $Y_{out}$ is output size, *p* is padding size, *k* kernel size, and *s* is stride. The next layer reduces the dimension to 56 × 56 with filter size 3 × 3. The combination of inception module creates a bottleneck structure for GoogleNet. The output layers are removed and features are transferred to a differential entropy-based selection of features.

As the SEED dataset consist of positive, negative and neutral classes by performing five trials on 15 subjects. The EEG collected while using only 26 channels is considered for each subject. These 26 images are made for each of the 26 individuals EEG signals using time-frequency representation with 1000 dimensions. The pre-trained weights are inherited to reduce the training time and initiated for EEG signal classification. The loss function for the proposed model is given as,

(9) $$Loss\left(r,\delta \right)=-\sum_{i=1}^{n}r_{i}log\left(\delta_{i}\right),$$

where *r* is 1 for true positive; otherwise, *r* is 0; δ is the probability of category *i* predicted by softmax.

### 3.3. Differential Entropy-Based Features Selection

To reduce the data dimension without sacrificing the performance of emotion detection model, a decision criteria that estimates the percentage of correlation with whole set of features with the subset of selected features. The correlation benefits in selecting high information features. Differential entropy (DE) divides the high information features from low information features. Due to this division of high and low information features, we find the boundary of uncertainty information. DE provides differences between sections created by subsets of features and all features from spectrum images [41]. Significantly, various essential characteristics can be derived from this difference measurement. The aim is to find a subset with similar properties and information as the original input data. DE feature reduction is similar to logarithm energy spectrum in a given frequency domain. The input EEG signal comprises of 1000 data points creating a spectrum of frequency. The filters results in multiple frequency bands: δ (1–4 Hz), θ (4–8 Hz), α (8–13 Hz), and β (13–30 Hz) from each EEG channel.

Suppose (U,A) is a collection of features system, considering *U* as a set of spectrum features, and *A* as a set of features. The decision table can be represented as (U,C∪D) is the collective features, for C∪D=A,C∩D=∅, *C* is non-empty set of conditional features, and *D* is the decision attributes, respectively. For every P⊆C, the DE feature vectors of *P* with respect to *C* is defined as;

(10) $$E\left(P|U⊕C\right)=-\frac{1}{U}\sum_{x\epsilon U}log_{2}\frac{\left|{\left[x\right]}_{C}\cap {\left[x\right]}_{P}\right|}{\left|{\left[x\right]}_{P}\right|}.$$

The information differentiation of *P* in the whole subset of (U,C∪D) is in fact the number of ${\left[x\right]}_{c}$ consisting of ${\left[x\right]}_{p}$. The differentiation represents the measure of boundary between feature subset *P* and entire set *C*.

The ${\left[x\right]}_{C}$ and ${\left[x\right]}_{P}$ are the corresponding indistinguishable relation determined for *P* and similarly for *C*. It must be noted that for any *P*⊆*C* and *x*ϵ*U*, is ${\left[x\right]}_{C}$⊆${\left[x\right]}_{P}$ and Equation (10) can be simplified as;

(11) $$E\left(P|U⊕C\right)=-\frac{1}{U}\sum_{x\epsilon U}log_{2}\frac{\left|{\left[x\right]}_{C}\right|}{\left|{\left[x\right]}_{P}\right|}.$$

Considering these formulations, it is observed that E(P|U⊕C) presents the the differentiation between U/P and U/C based on their actual ability. Additionally, it shows the importance of subset *P* in comparison to entire original feature set *C*. In fact, the higher the E(P|U⊕C), the higher the difference between feature sets *P* in the depiction of *C*. Specifically, if E(P|U⊕C)=0, then U/P=U/C implies that both subset feature U/P and whole feature set U/C have equal knowledge. Only features with high entropy are selected to process in BoDF. Reducing the low entropy-based features can reduce features dimensions without degrading performance and improve simulation time resulting in a lightweight model.

### 3.4. Bag of Deep Features (BoDF)

BoDF is applied to minimize the collection of redundant features in classification. The reduced features can decrease feature size and the time required to train the backbone model. In general, many features have huge simulation time and high computational complexity. It is observed that using only 8 to 12 channels has a detrimental effect on the performance of the backbone model. The BoDF model comprises two stages; first, the Google-Net and differential entropy features are extracted from each EEG channel and are combined into a single vector. In the second stage, the cumulative set of all 26 features is clustered with the K-mean clustering method. The K-means clustering accumulates similar features into one region that represents the vocabulary employed in the further extraction of the final feature vector. The vocabulary set then generates the similarity histogram of raw feature vectors. The histogram is achieved using the euclidean distance of the vocabulary and raw feature vectors. The frequency of vocabulary features in the raw data denotes the final representation of the EEG data. For the SEED dataset the features of 17,550 × 1000 are clustered to 255 × 25 features vectors. Determining the number of clusters *k* is crucial for estimating decision boundary. The optimal values for choosing the suitable number of clusters are obtained through the hit and trial method. The 255 × 25 for each class feature vector is termed as its vocabulary. In the second phase, the vocabulary histogram is estimated. The comparison is performed by the vocabulary of each feature vector of 255 × 25 with the EEG dataset of all channels. In SEED dataset, features of 255 × 25 were compared with 26 channels features, and the occurrence frequency is computed. The size of the histogram feature is 225 × 25, along with the attribute value of the histogram between 0 and 30, which reduces the large feature size. The histogram features obtain the frequency of optimal features for each class. These features are collectively passed to classifiers. Multiple classifiers are proposed to provide a comparison and select a better classifier. Since the classification layer of the GoogleNet model is removed, SVM, ensemble, tree, and KNN classifiers are used as a classification layer.

### 3.5. Dataset Description

An EEG signals dataset was collected by Shanghai Jiaotong University (SJTU) for examining and analysis of the EEG signals for emotion detection known as SEED dataset [42]. The dataset contains EEG signals that are taken when human beings are watching different movies. In total, the data were collected from 15 participants (eight females and seven males). They were subjected to 15 movie clips containing various scenes to invoke a human being’s different emotions. These emotions of these people are noted and labeled as positive, negative, and neutral based on happy, sad, and normal mode, respectively. Table 2 shows the names of clips from few movies and the result of invoking emotion. The following protocols are satisfied when collecting the dataset:

- The emotion detection was recorded for short clips in order to avoid the unnatural behavior of the human.
- The video explains the scenario of itself.
- Only one emotion was recorded at the time of watching the video.

**Figure 5:**
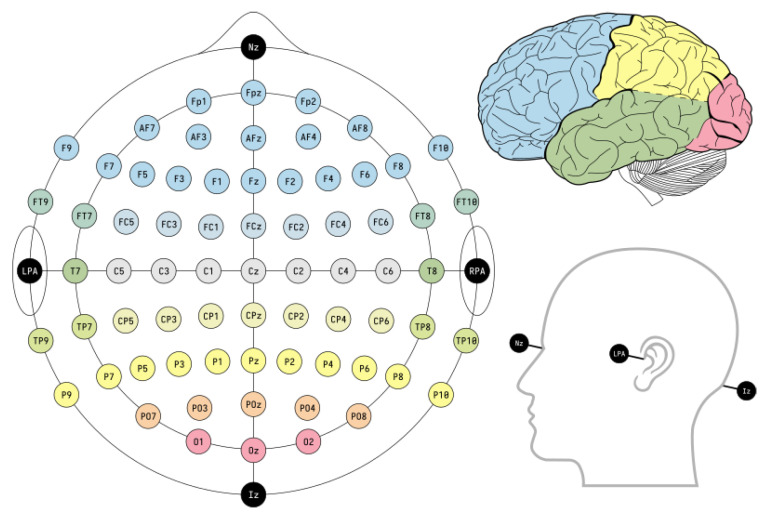
The placement chart of the International 10–20 System and allocation of all 62 channels.

The human emotional data were collected according to the international standard of the 10–20 system. The electrodes are placed on the head of the human beings to record EEG exams, poly-monograph, sleep, and study. These standards ensure the testing, compiling, and analysis of data for scientific purposes. To measure EEG signals, the electrodes are placed on anatomical landmarks on the human skull. The distance between the electrodes is 10% or 20% of the total front-back and right-left distances on the skull therefore, the system is known as the 10–20 system. The placement of electrodes is presented in Figure 5.

## 4. Results

Extensive experiments were performed through different deep learning models with different handcraft feature extraction classifiers. The performance is evaluated on the SEED dataset that consists of different channels for three classes. Only 26 channels are considered, to reduce cumbersome features while maintaining comparable accuracy. The performance is evaluated with multiple backbone models that are GoogleNet, AlexNet, ResNet-50, ResNet-101, and Inception-ResNet V2 with a combination of a different number of channels. Only the channels with best results on every backbone models are presented.

### 4.1. Performance Evaluation on Different Deep Learning Models

**Figure 6:**
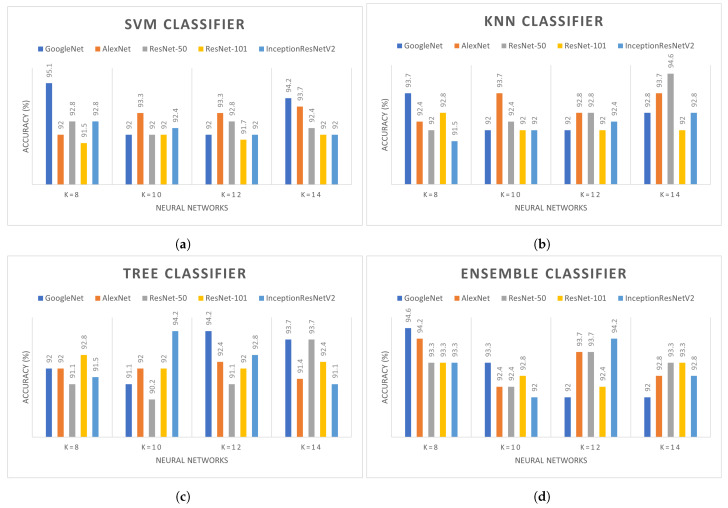
Comparison of different deep learning with (**a**) SVM, (**b**) KNN, (**c**)Tree, (**d**) Ensemble classifier.

Multiple experiments were performed on different deep learning models in combination with the handcraft classifiers. The last classification layers are removed from the backbone models and evaluated on different handcraft classifiers such as ensemble and decision trees. It is compulsory to evaluate each deep learning model with different values of *k* due to their direct influence on the overall accuracy. The backbone models such as GoogleNet, AlexNet, ResNet-50, ResNet-101, and Inception V2, are combined with SVM, KNN, ensemble, and decision trees with k=(8,10,12,14) in clustering. The effect of different *k* values on SVM, KNN, ensemble, and decision tree classifiers with multiple deep learning models are presented in Figure 6. The performance of GoogleNet with the SVM classifier is comparatively better than other backbone models. As shown in Figure 6, the highest accuracy of SVM is 95.1% and 94.2% achieved with *k* = 8 and 14. However, accuracy achieved with AlexNet is 93.3% and 93.7% for *k* = 10, 12, and 14. With decision tree classifiers, GoogleNet and Inception-ResNetV2 have similar accuracy when *k* values are set to 12 and 10, respectively. Both of these models achieved 94.2% accuracy while in all other models the accuracy is between 91% and 93%. AlexNet and ResNet-50 achieve 94.6% with the KNN classifier. While using ensemble networks, the performance of GoogleNet is higher among all. However, as shown in Figure 6, the overall performance of GoogleNet is better with all *k* values in comparison to other backbone models. Table 3 presents the performance evaluation of different backbone models with the minimum number of channels considering different kernels in classifiers. The cubic kernel in SVM has the highest accuracy with 96.7% among all kernels using only 26 channels. The minimum number of channels reduces computation and cumbersome calculation. Subspace KNN kernel in ensemble classifier achieved the second highest accuracy of 96.2% followed by tree and KNN with 95.8% and 95.3%, respectively. Multiple experiments were performed with the different number of channels on different kernels; however, only the best results are shown in Table 3.

### 4.2. Comparison with State-of-the-Art Methods

The proposed method is compared with the state-of-the-art methods for emotion detection in Table 4. The comparison methods are motor imagery classification (MIC) [25], emotion recognition through wavelet transforms (ER-WTF) [31], empirical mode decomposition (EMD) [23], spatio-temporal recurrent neural network (STRNN) [14], cross subject (CS) [15], Evolutionary computation (EC) [39], and multi-model (MM) [38]. The proposed DEFS method achieved better performance on all classifiers with GoogleNet backbone in comparison to state-of-the-art methods. Specifically, the overall accuracy on SEED dataset is improved up to 96.7%, 95.3%, 95.8%, and 96.2% for SVM, KNN, tree, and ensemble classifiers. ER-WTF [31] uses a minimum number of dataset channels while STRNN [14] uses all 62 channels but the accuracy is still not satisfactory. Despite using fewer channels on SEED dataset, the proposed method achieved satisfactory performance for detecting various emotions. Compared to the SVM classifier of MMT [38], the proposed method’s accuracy using SVM, KNN, ensemble, and tree classifier is improved up to 2.9%, 2.4%, 2.4% and 2.0%, respectively. This indicates the effectiveness of the proposed method in accurate emotion recognition and classification.

## 5. Conclusions

In this article, a novel differential entropy-based feature selection technique is proposed to address the correct detection of emotion. Using GoogleNet as a backbone model can provide rich features using fewer channels. A guide to extracting multiple features based on histograms and clusters of similar features is added to the backbone model to improve the learning process. Different machine learning classifiers are added with multiple kernels to evaluate accurate detection. Extensive experiments on the SEED dataset show that proposed emotion detection provides satisfactory results. Nevertheless, emotion detection of EEG signals still requires more improvement. Further evaluation is needed through lightweight deep learning models and handcraft classifiers to obtain excellent detection accuracy. EEG-based emotion detection requires multi-disciplinary knowledge such as neuroscience, psychology, engineering, and computer science. Multi-model learning can be implemented to surpass the performance of current algorithms, or further discovery in psychology and neuroscience is required to strengthen detection accuracy.

## Figures and Tables

**Table 1:** Comparison of our proposed work with the previous studies.

Methods	Features	Dataset	No. of Channels	Classifier
MIC [25]	MFM	DEAP	18	CapsNet
ER-WTF [31]	MFCC	SEED		SVM
				Random Forest
		DEAP	6	Random Forest
EMD [23]	MEMD	DEAP	12	ANN
				KNN
STRNN [14]	STRNN	SEED	62	CNN
CS [15]	RFE	SEED	18	SVM
		DEAP	12	SVM
EC [39]	DE	DEAP	32	PNN
MM [38]	BODF	SEED	62	SVM
				KNN
		DEAP	32	SVM
				KNN
Our Work	DEFS	SEED	26	SVM
				KNN
				Tree
				Ensemble

**Table 2:** Description of Clips that invoke Positive, Negative, Neutral Emotions.

No.	Emotion Label	Clips from Movie Source
1	Negative	Tangshan Earthquake
2	Negative	1942
3	Positive	Lost in Thailand
4	Positive	Flirting scholar
5	Positive	Just another Pandora’s Box
6	Neutral	World Heritage in China

**Table 3:** Analysis of accuracy following channels reduction of various deep learning models.

Neural Networks	Channels	Classifiers	Kernals	Accuracy (%)
GoogleNet	26	SVM	Cubic	96.7
		kNN	Fine	95.3
		Tree	Medium	95.8
		Ensemble	Subspace KNN	96.2
AlexNet	28	SVM	Fine Gaussian	95.3
		kNN	Weighted	96.2
		Tree	Medium/Fine	94.0
		Ensemble	Subspace KNN	95.8
Resnet-50	40	SVM	Fine Gaussian	94.4
		kNN	Weighted	96.2
		Tree	Medium/Fine	95.3
		Ensemble	Subspace KNN	95.3
Resnet-101	29	SVM	Fine Gaussian	94.0
		kNN	Weighted	94.4
		Tree	Medium/Fine	94.4
		Ensemble	Bagged Trees	94.9
InceptionresnetV2	32	SVM	Cubic	94.4
		kNN	Weighted/Fine	94.4
		Tree	Medium/Fine	95.8
		Ensemble	Subspace KNN	95.8

**Table 4:** Comparison of our proposed work with the previous work on emotion detection.

Methods	Features	Dataset	No. of Channels	Classifier	Accuracy (%)
MIC [25]	MFM	DEAP	18	CapsNet	68.2
ER-WTF [31]	MFCC	SEED	6	SVM	83.5
				Random Forest	72.07
		DEAP	6	Random Forest	72.07
EMD [23]	MEMD	DEAP	12	ANN	75
				KNN	67
STRNN [14]	STRNN	SEED	62	CNN	89.5
CS [15]	RFE	SEED	18	SVM	90.4
		DEAP	12	SVM	60.5
EC [39]	DE	DEAP	32	PNN	79.3
MM [38]	BODF	SEED	62	SVM	93.8
				KNN	91.4
		DEAP	32	SVM	77.4
				KNN	73.6
Our Work	DEFS	SEED	26	SVM	96.7
				KNN	95.3
				Tree	95.8
				Ensemble	96.2

## Data Availability

The SEED dataset can found at following link: Available online: https://bcmi.sjtu.edu.cn/home/seed/seed.html (accessed on 30 May 2022).

## Abbreviations

The following abbreviations are used in this manuscript:

BoDF	Bag of Deep Features
CS	Cross Subject
CSP	Common Spatial Patterns
CWT	Wavelet Transform
DE	Differential Entropy
DECS	Differential Entropy-Based Channel Selection
DWT	Discrete Wavelet Transform
EC	Evolutionary Computation
EEG	Electroencephalogram signals
EMD	Empirical Mode Decomposition
EMG	Electromyography
ER-WTF	Emotion Recognition Through Wavelet Transforms
fMRI	Functional Magnetic Resonance Imaging
KNN	K-nearest Neighbours
MIC	Motor Imagery Classification
MM	Multi-model learning
1D	One-Dimensional
ReLU	Rectified Linear Activation
RFE	Recursive Emotion Feature Elimination
SJTU	Shanghai Jiaotong University
STRNN	Spatial-Temporal Recurrent Neural Networks
SVM	Support Vector Machine
STFT	Short-Time Fourier transform
2D	two-dimensional
WPD	Wavelet Packet Decomposition
